# Supplementary material for: Application of Bioelectrical Impedance Analysis (BIA) to Assess Carcass Composition and Nutrient Retention in Rabbits from 25 to 77 Days of Age
Source: Animals (Basel). 2022 Oct 25;12(21):2926. doi: 10.3390/ani12212926 (PMC9654842; doi:10.3390/ani12212926)
Supplement: Supplementary file 1 [file animals-12-02926-s001.zip › animals-1919970-supplementary.pdf]

Table S1. Average values of resistance (Rs), reactance (Xc), impedance (Z), distance between electrodes (D) , animal body length (L) and Body Weight (BW) of the validation group rabbits (n=5) at 25, 35, 49, 63 and 77 days of age

| Age, d | [1] | Rs, Ω | Xc, Ω | Z, Ω | D, cm | L, cm | BW, g |
|---|---|---|---|---|---|---|---|
| 25 | Mean | 121 | 16.4 | 122 | 10.1 | 18.0 | 370 |
| | Min | 89.5 | 10.0 | 90.2 | 6.00 | 12.0 | 196 |
| | Max | 169 | 23.5 | 170 | 12.5 | 22.0 | 507 |
| | SD | 21.3 | 4.06 | 21.5 | 1.64 | 2.79 | 105 |
| 35 | Mean | 92.9 | 16.8 | 94.4 | 10.5 | 21.0 | 634 |
| | Min | 67.5 | 12.0 | 68.6 | 7.25 | 18.0 | 405 |
| | Max | 115 | 19.5 | 116 | 13.0 | 27.0 | 1129 |
| | SD | 13.3 | 1.97 | 13.3 | 1.50 | 2.30 | 178 |
| 49 | Mean | 93.7 | 21.9 | 96.2 | 12.7 | 27.4 | 1220 |
| | Min | 81.5 | 18.5 | 83.6 | 10.7 | 26.0 | 926 |
| | Max | 109 | 27.0 | 111 | 14.5 | 30.0 | 1436 |
| | SD | 10.3 | 2.48 | 10.2 | 1.20 | 1.43 | 178 |
| 63 | Mean | 101 | 24.5 | 104 | 16.3 | 30.5 | 1912 |
| | Min | 54.5 | 20.0 | 60.0 | 14.0 | 25.0 | 1573 |
| | Max | 128 | 29.0 | 131 | 18.5 | 35.0 | 2228 |
| | SD | 15.6 | 2.45 | 15.3 | 1.39 | 2.02 | 210 |
| 77 | Mean | 63.0 | 16.2 | 65.0 | 18.5 | 33.8 | 2930 |
| | Min | 45.0 | 11.0 | 46.3 | 14.0 | 31.0 | 2090 |
| | Max | 95.5 | 25.5 | 98.9 | 21.0 | 39.0 | 3260 |
| | SD | 14.6 | 3.89 | 15.1 | 2.04 | 2.38 | 321 |

[1]Min: minimum value; Max: maximum value; SD: standard deviation

Table S2. Average values of resistance (Rs), reactance (Xc), impedance (Z), distance between electrodes (D) , animal body length (L) and Body Weight (BW) of the validation group rabbits (n=5) at 25, 35, 49, 63 and 77 days of age.

| **Age, d** | [1] | **Rs, Ω** | **Xc, Ω** | **Z, Ω** | **D, cm** | **L, cm** | **BW, g** |
|---|---|---|---|---|---|---|---|
| 25 | Mean | 130 | 18.5 | 132 | 10.5 | 17.2 | 378 |
| | Min | 105 | 14.5 | 106 | 9.00 | 16.0 | 361 |
| | Max | 151 | 24.5 | 153 | 12.7 | 19.0 | 392 |
| | SD | 21.5 | 3.94 | 21.7 | 1.38 | 1.30 | 135 |
| 35 | Mean | 95.8 | 18.9 | 97.7 | 10.9 | 21.3 | 644 |
| | Min | 80.5 | 14.5 | 81.8 | 9.50 | 20.0 | 630 |
| | Max | 114 | 24.0 | 116 | 12.5 | 23.0 | 654 |
| | SD | 14.2 | 3.54 | 14.5 | 1.15 | 1.40 | 90.1 |
| 49 | Mean | 89.2 | 22.0 | 91.9 | 14.2 | 21.3 | 1,291 |
| | Min | 87.5 | 21.5 | 90.1 | 13.7 | 20.0 | 1,283 |
| | Max | 91.0 | 22.5 | 93.7 | 14.7 | 23.0 | 1,299 |
| | SD | 2.47 | 0.71 | 2.57 | 0.71 | 1.39 | 113 |
| 63 | Mean | 99.3 | 24.3 | 102 | 15.7 | 30.6 | 1,938 |
| | Min | 80.5 | 20.5 | 83.1 | 14.5 | 29.0 | 1,911 |
| | Max | 113 | 27.0 | 116 | 16.5 | 33.0 | 1,970 |
| | SD | 15.2 | 3.06 | 15.4 | 0.86 | 1.52 | 243 |
| 77 | Mean | 75.2 | 14.5 | 76.7 | 14.2 | 27.7 | 2,985 |
| | Min | 57.5 | 11.0 | 59.7 | 13.7 | 25.5 | 2,969 |
| | Max | 84.5 | 17.0 | 85.6 | 14.7 | 30.0 | 3,020 |

| | | | | | | |
|---|---|---|---|---|---|---|
| | SD | 12.3 | 2.64 | 11.7 | 0.71 | 3.18 | 241 |

[1]Min: minimum value; Max: maximum value; SD: standard deviation

Table S3. Correlation Matrix between bioelectrical impedance analysis and carcass composition expressed as a percentage of calibration group rabbits ($n$=75)[1].

| | Xc, Ω | Rs, Ω | Water, % | Protein, %DM | Fat, %DM | Ash, %DM | Energy, kJ/100 g DM | BW, g | Age, d | L, cm | D, cm | Carcass yield, % |
|---|---|---|---|---|---|---|---|---|---|---|---|---|
| Xc, Ω | 1 | 0.35** | −0.22 | 0.01 | 0.24* | −0.10 | 0.18 | 0.16 | 0.08 | −0.05 | 0.30** | 0.84**** |
| Rs, Ω | | 1 | 0.55**** | 0.40*** | −0.44**** | 0.54**** | −0.55**** | −0.63**** | −0.63**** | −0.04 | −0.58**** | 0.81**** |
| Water, % | | | 1 | 0.88**** | −0.97**** | 0.96**** | −0.98**** | −0.98**** | −0.97**** | −0.17 | −0.94**** | 0.80**** |
| Protein, %DM | | | | 1 | −0.93**** | 0.88**** | −0.88**** | −0.86**** | −0.89**** | −0.29* | −0.75**** | −0.54**** |
| Fat, %DM | | | | | 1 | −0.93**** | 0.95**** | 0.95**** | 0.95**** | 0.18 | 0.89**** | −0.36**** |
| Ash, %DM | | | | | | 1 | −0.99**** | −0.94**** | −0.95**** | −0.19 | −0.85**** | 0.83**** |
| Energy, kJ/100 g DM | | | | | | | 1 | 0.97**** | 0.97**** | 0.16 | 0.90**** | 0.65**** |
| BW, g | | | | | | | | 1 | 0.99**** | 0.12 | 0.96**** | 0.84**** |
| Age, d | | | | | | | | | 1 | 0.21 | 0.93**** | 0.84**** |
| L, cm | | | | | | | | | | 1 | 0.05 | 0.83**** |
| D, cm | | | | | | | | | | | 1 | −0.41**** |
| Carcass yield, % | | | | | | | | | | | | 1 |

[1]Xc = reactance; Rs = resistance; L = body length; D = distance between internal electrodes. *$P$ < 0.05; **$P$ < 0.01; ***$P$ < 0.001; ****$P$ < 0.0001.

Table S4. Regression coefficients and SE for equations determined by MLR for predicting carcass water and protein content expressed as DM percentage and grams ($n$=75)

| Dependent variable | Independent variable[1] | Estimate | SE | $P$-value | $R^2$ | rsd[2] | CV | $P$m[3] | $Cp$[4] |
|---|---|---|---|---|---|---|---|---|---|
| Water, % | Intercept | 68.8 | 3.31 | <0.001 | 0.79 | 1.62 | 2.35 | <0.001 | 2.29 |
| | $BW^2$ | $-9.55 \times 10^{-7}$ | $1.44 \times 10^{-7}$ | <0.001 | | | | | |
| | L | −0.16 | 0.0805 | 0.05 | | | | | |
| | Xc | 0.68 | 0.3648 | 0.06 | | | | | |
| | $Xc^2$ | −0.019 | 0.0088 | 0.04 | | | | | |
| | vol1 | 0.06 | 0.2706 | 0.05 | | | | | |
| Water, g | Intercept | 2.66 | 2.24 | 0.91 | 0.99 | 38.6 | 7.23 | <0.001 | 0.92 |
| | Age | 1.68 | 1.18 | 0.16 | | | | | |
| | BW | 0.21 | 0.043 | <.001 | | | | | |
| | $BW^2$ | $3.80 \times 10^{-5}$ | $8.38 \times 10^{-6}$ | <.001 | | | | | |
| | vol2 | 16.7 | 5.39 | 0.003 | | | | | |
| Protein, %DM | Intercept | 75.2 | 5.19 | <0.001 | 0.68 | 3.20 | 5.53 | <0.001 | 2.39 |
| | BW | 0.0051 | 0.0018 | 0.006 | | | | | |
| | $BW^2$ | $-3.22 \times 10^{-6}$ | $5.71 \times 10^{-7}$ | <0.001 | | | | | |
| | Rs | −0.26 | 0.10 | 0.01 | | | | | |
| | $Rs^2$ | $9.75 \times 10^{-4}$ | $4.94 \times 10^{-4}$ | 0.05 | | | | | |
| Protein, g | Intercept | 41.4 | 27.0 | 0.13 | 0.99 | 13.2 | 9.16 | <0.001 | 5.32 |
| | BW | 0.073 | 0.0098 | <0.001 | | | | | |
| | $BW^2$ | $8.84 \times 10^{-6}$ | $2.73 \times 10^{-6}$ | 0.002 | | | | | |
| | Xc | −4.72 | 2.77 | 0.09 | | | | | |
| | $Xc^2$ | 0.13 | 0.069 | 0.07 | | | | | |
| | vol2 | 5.77 | 2.01 | 0.005 | | | | | |

[1]L: body length; Xc: reactance; Rs: resistance; Z: impedance; D: distance between internal electrodes; vol1: $D^2$/Rs; vol2: $D^2$/Z

[2]rsd = residual SD. [3]$P$m = probability of the model. [4] Mallows' Cp statistics [30].

Table S5. Regression coefficients and SE of the equations determined by MLR for predicting carcass ash and fat content expressed as DM percentage and grams ($n$=75)

| Dependent variable | Independent variable[1] | Estimate | SE | $P$-value | $R^2$ | rsd[2] | CV | $P$m[3] | $Cp$[4] |
|---|---|---|---|---|---|---|---|---|---|
| Ash, %DM | Intercept | 7.31 | 3.72 | 0.05 | 0.66 | 1.83 | 11.9 | <0.001 | 2.66 |
| | Age | 0.15 | 0.050 | 0.004 | | | | | |
| | BW | −0.0053 | $9.63 \times 10^{-4}$ | <0.001 | | | | | |
| | Xc | 0.93 | 0.37 | 0.01 | | | | | |
| | $Xc^2$ | −0.02 | 0.009 | 0.009 | | | | | |
| Ash, g | Intercept | −7.09 | 3.62 | 0.05 | 0.96 | 5.02 | 14.1 | <0.001 | -1.19 |
| | Age | 0.19 | 0.11 | 0.10 | | | | | |
| | BW | 0.02 | 0.003 | <0.001 | | | | | |

| | | | | | | | | | |
|---|---|---|---|---|---|---|---|---|---|
| | Rs² | 1.97 x $10^{-4}$ | 1.59 x $10^{-4}$ | 0.22 | | | | | |
| | vol2 | 1.45 | 0.66 | 0.03 | | | | | |
| Fat, %DM | Intercept | 16.9 | 1.32 | <0.001 | 0.76 | 3.52 | 14.1 | <0.001 | 4.61 |
| | BW² | 2.41 x $10^{-6}$ | 2.85 x $10^{-7}$ | <0.001 | | | | | |
| | Xc² | 0.00796 | 0.002 | 0.001 | | | | | |
| | vol1 | −28.9 | 12.6 | 0.02 | | | | | |
| | vol2 | 28.8 | 13.1 | 0.03 | | | | | |
| Fat, g | Intercept | 9.73 | 4.02 | 0.02 | 0.95 | 19.0 | 24.0 | <0.001 | 0.27 |
| | BW² | 2.94 x $10^{-5}$ | 1.50 x $10^{-5}$ | <0.001 | | | | | |
| | vol1 | −97.4 | 66.3 | 0.15 | | | | | |
| | vol2 | 93.6 | 68.9 | 0.18 | | | | | |

[1]Xc: reactance; Rs: resistance; Z: impedance; D: distance between internal electrodes; vol1: $D^2$/Rs; vol2: $D^2$/Z
[2]rsd = residual SD. [3]*P*m = probability of the model. [4] Mallows' Cp statistics [30].

Table S6. Regression coefficients and SE of the equations determined by MLRfor predicting carcass energy content as kJ/100g DM and MJand carcass yield expressed as a percentage (*n*=75).

| Dependent variable | Independent variable[1] | Estimate | SE | *P*-value | R² | rsd[2] | CV | *P*m[3] | *Cp*[4] |
|---|---|---|---|---|---|---|---|---|---|
| Energy, kJ/100 g DM | Intercept | 2532 | 207 | <0.001 | 0.82 | 102 | 4.59 | <0.001 | 9.90 |
| | Age | −9.21 | 2.80 | 0.002 | | | | | |
| | BW | 0.39 | 0.05 | <0.001 | | | | | |
| | Xc | −51.3 | 20.5 | 0.01 | | | | | |
| | Xc² | 1.47 | 0.53 | 0.007 | | | | | |
| Energy, MJ | Intercept | 2.42 | 1.60 | 0.13 | 0.98 | 0.83 | 13.2 | <0.001 | -0.99 |
| | BW² | 1.82 x $10^{-6}$ | 3.11 x$10^{-8}$ | <0.001 | | | | | |
| | Xc | −0.19 | 0.17 | 0.27 | | | | | |
| | Xc² | 0.006 | 0.004 | 0.19 | | | | | |
| Carcass yield, % | Intercept | 50.02 | 2.61 | <0.001 | 0.50 | 4.73 | 8.78 | <0.001 | 6.56 |
| | BW² | 1.79 x $10^{-6}$ | 2.20 x $10^{-7}$ | <0.001 | | | | | |
| | Xc | −0.44 | 0.126 | <0.001 | | | | | |
| | Rs² | 7.41 x $10^{-4}$ | 1.53 x $10^{-4}$ | <0.001 | | | | | |

[1]Xc: reactance; Rs: resistance [2]rsd = residual SD. [3]*P*m = probability of the model.[4] Mallows' Cp statitics [30].
